# Natural Antimicrobial Agents from Algae: Current Advances and Future Directions

**DOI:** 10.3390/ijms252111826

**Published:** 2024-11-04

**Authors:** Antonio Zuorro, Roberto Lavecchia, Jefferson E. Contreras-Ropero, Janet B. García Martínez, Crisóstomo Barajas-Ferreira, Andrés F. Barajas-Solano

**Affiliations:** 1 Department of Chemical Engineering, Materials and Environment, Sapienza University, Via Eudossiana 18, 00184 Roma, Italy; roberto.lavecchia@uniroma1.it; 2 Department of Environmental Sciences, Universidad Francisco de Paula Santander, Av. Gran Colombia No. 12E-96, Cucuta 540003, Colombia; jeffersoneduardocr@ufps.edu.co (J.E.C.-R.); janetbibianagm@ufps.edu.co (J.B.G.M.); andresfernandobs@ufps.edu.co (A.F.B.-S.); 3 School of Chemical Engineering, Universidad Industrial de Santander, Cra 27, Calle 9, Bucaramanga 680006, Colombia; cbarajas@uis.edu.co

**Keywords:** algae, cyanobacteria, antimicrobial, bioactive compounds, biosynthesis

## Abstract

Infectious diseases have significantly shaped human history, leading to significant advancements in medical science. The discovery and development of antibiotics represented a critical breakthrough, but the rise of antibiotic-resistant pathogens now presents a serious global health threat. Due to the limitations of current synthetic antimicrobials, such as toxicity and environmental concerns, it is essential to explore alternative solutions. Algae, particularly microalgae and cyanobacteria, have emerged as promising sources of bioactive antimicrobial compounds. This review provides a comprehensive analysis of the antimicrobial properties of algal-derived compounds, including polysaccharides, fatty acids, and phenols, which have shown effectiveness against multi-drug-resistant bacteria. A co-occurrence bibliometric analysis using VOSviewer highlighted five key research clusters: antibiotic resistance, algal extracts, biosynthesis, water treatment, and novel pharmacological compounds. Furthermore, the primary mechanisms of action of these bioactive compounds, such as the inhibition of protein synthesis and cell membrane disruption, were identified, demonstrating their potential against both common and multi-resistant pathogens. Future research should prioritize optimizing algal biomass production, utilizing genetic and metabolic engineering, and creating innovative delivery systems to enhance the efficient production of bioactive compounds.

## 1. Introduction

Throughout history, infectious diseases have profoundly impacted humanity, influencing the progress of medical science and the very structure of societies. Notable pandemics such as the Black Death in the 14th century, which claimed nearly a third of Europe’s population, and the 1918 Spanish flu, responsible for approximately 50 million deaths, serve as stark reminders of how these outbreaks have shaped human history [1]. Efforts such as the global eradication of smallpox through vaccination and the ongoing struggle against HIV/AIDS highlight the continued significance of infectious disease research [2]. These epidemiological disasters have exposed our susceptibility to pathogens, driving significant advancements in preventive and therapeutic medicine [3].

The discovery of antibiotics stands as a pivotal moment in medical history. In 1928, Alexander Fleming identified penicillin, a compound produced by the fungus Penicillium notatum, which disrupts bacterial cell wall synthesis, destroying bacteria [1]. This breakthrough, followed by the development of other antibiotics like streptomycin and tetracycline, transformed the treatment of bacterial infections and significantly reduced mortality rates [2]. The introduction of antibiotics saved countless lives and made previously high-risk medical procedures safer.

We currently face a crisis of microbial resistance to commercial antibiotics, a significant problem threatening to reverse the advances that antibiotics have achieved. The causes of this crisis include the excessive and inappropriate use of antibiotics in human and veterinary medicine and the lack of development of new antibiotics. According to the World Health Organization (WHO), approximately 700,000 people die each year due to drug-resistant infections. This figure could rise to 10 million by 2050 if adequate measures are not taken [1]. The Centers for Disease Control and Prevention (CDC) have also warned about the devastating impact this crisis could have on global public health [3].

The synthetic antimicrobials currently produced have several limitations and adverse effects. These issues include toxicity, disruption of the human microbiome, and adverse environmental health impacts. Studies have shown that these medications can cause liver and kidney damage and that alterations in the gut microbiome can lead to various health complications, including secondary infections and metabolic disorders [4]. The microbiome disruption has mainly been linked to increased susceptibility to infections and poor recovery from chemically induced injuries [5]. Moreover, exposure to antimicrobials can modify the microbiota, resulting in microbial resistance and other long-term adverse effects [4].

Exploring alternative sources of antimicrobials is crucial to address microbial resistance and ensure effective and sustainable treatments. Recent research has evaluated the antimicrobial potential of natural sources such as medicinal plants, fungi, and marine products. For example, studies have demonstrated that extracts from the neem tree (*Azadirachta indica*) and turmeric (*Curcuma longa*) possess significant antimicrobial properties. One study found that neem extracts have notable antimicrobial activity against *Streptococcus mutans* and *Candida albicans*, showing inhibition zones comparable to chlorhexidine [6]. Another study highlighted that turmeric exhibits antimicrobial activity against resistant bacteria such as *Escherichia coli* and *Staphylococcus aureus*, attributing this efficacy to the curcuminoids present in the plant [7].

An up-and-coming emerging source of antimicrobial compounds is algae, including both microalgae and cyanobacteria. These organisms have proven to be rich sources of bioactive compounds with antimicrobial properties. The secondary metabolites produced by these species, including polysaccharides, fatty acids, phenols, terpenes, alkaloids, and halogenated compounds, have shown significant antimicrobial properties. For example, Eicosapentaenoic Acid, isolated from *Phaeodactylum tricornutum*, has demonstrated efficacy against multi-resistant bacteria, including methicillin-resistant *S. aureus* [8]. Cyanobacteria, such as *Arthorspira platensis*, have also shown activity against various bacterial and fungal pathogens [9].

The potential of microalgae and cyanobacteria is further reflected in their ability to be cultivated in large volumes in controlled bioreactors, ensuring sustainable and continuous production of bioactive compounds. Moreover, these organisms have the advantage of rapid growth and can be cultivated under various environmental conditions, making them more efficient in production than other natural organisms. Research has shown that extracts from macroalgae such as *Gracilaria corticata* and *Hydroclathrus clathratus* exhibit significant antimicrobial activity, inhibiting the growth of human pathogens [10]. The polyphenols from algae, particularly phlorotannins, also show high antimicrobial activity against Gram-positive and Gram-negative bacteria [11]. These characteristics and their high efficacy and low toxicity make compounds derived from microalgae and cyanobacteria a superior alternative to other natural antimicrobial sources [12].

The primary objective of this review is to provide a comprehensive and updated overview of the latest trends in the research and development of antimicrobial products derived from algae and cyanobacteria. Through bibliometric analysis and a review of the scientific literature, this review will address the antimicrobial properties of algae, the extraction and purification techniques used, potential biotechnological and clinical applications, and the challenges and future perspectives in this field. Additionally, the technological and methodological advances that have enabled a greater understanding and utilization of the bioactive properties of algae will be highlighted. This consolidation of knowledge aims to identify research areas that require more attention and suggest innovative approaches for exploiting algae as a source of new antimicrobial agents.

## 2. Results

### 2.1. Bibliometric Impact

**Figure 1:**
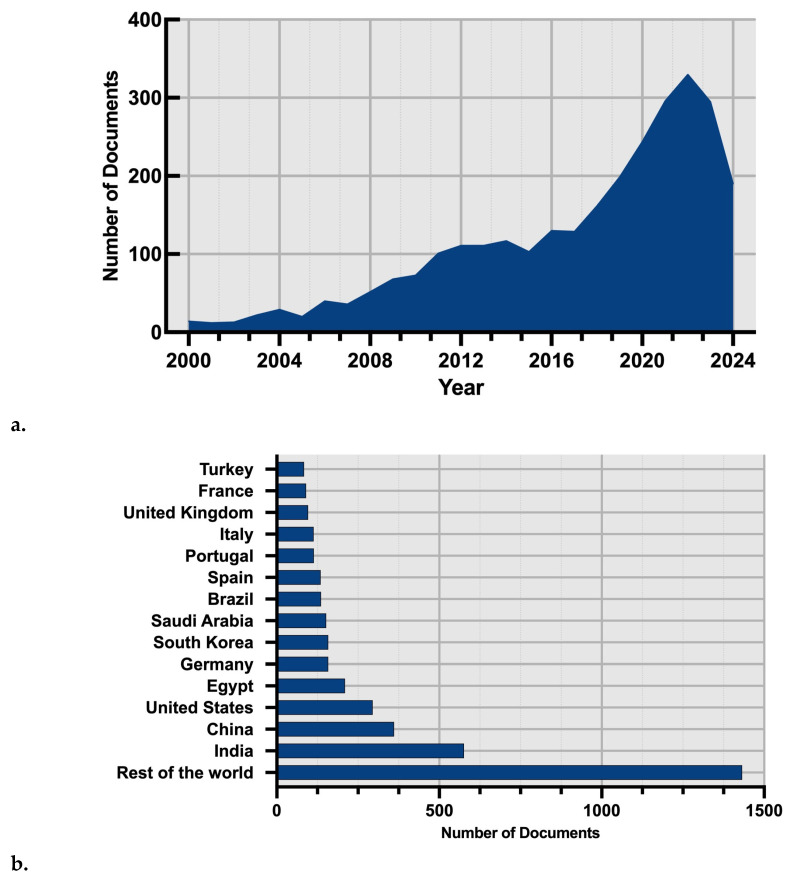
Number of documents published by year (**a**) and country (**b**) according to relevant scientific publications. Specific keywords were used: (Algae OR Meta Cyano-bacteria) and (antimicrobial OR antibiotic OR bioactive) between 2000 and 2024.

According to the bibliographic data obtained in Scopus, over the last 20 years, over 2897 scientific documents (2297 research papers and 600 review papers) have been published. The exponential growth in scientific documents (from the year 2000 to 2022) shows the immense interest from the scientific community toward understanding the chemical mechanism involved in the production and activity of algae-based antimicrobials (Figure 1a,b).

### 2.2. Co-Occurrence Analysis

**Figure 2:**
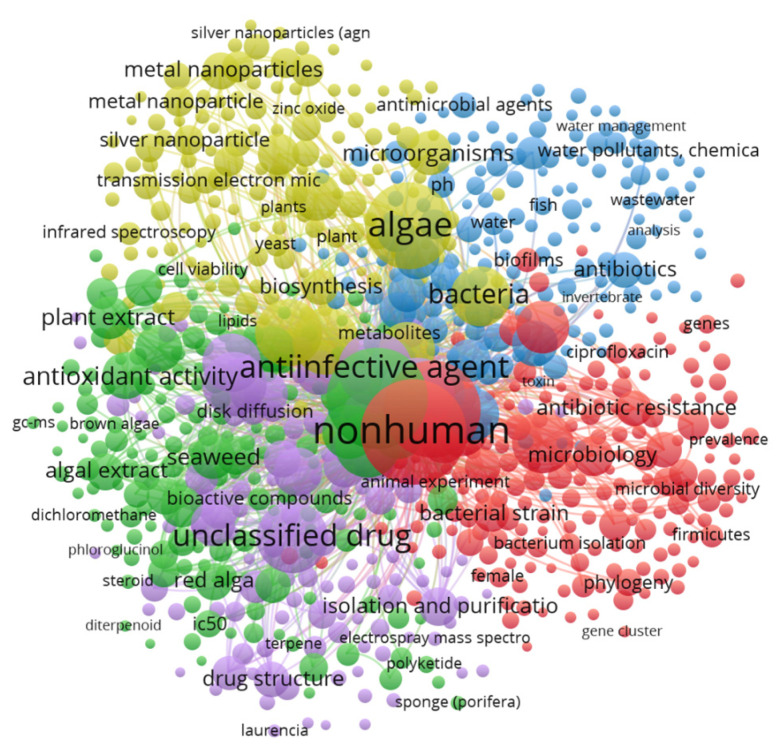
Map of Co-Occurrence of Terms in Algal Antimicrobials. according to relevant scientific publications with the specific keywords: (Algae OR Meta Cyano-bacteria) and (antimicrobial OR antibiotic OR bioactive) between 2000 and 2024.

The co-occurrence analysis performed using VOSviewer provides a detailed insight into the relationships between terms within the research corpus focused on algae and antimicrobials (Figure 2). This approach is crucial for identifying key research areas and the semantic interconnections between terms. The analysis revealed five main clusters, each representing a distinct research area, offering a deeper understanding of central themes and complex linguistic relationships within the research. The clusters are as follows:

- Red Cluster: This cluster focuses on the interaction between bacteria and antibiotics, including terms like “non-human”, “bacteria”, “antibiotics”, “antibiotic resistance”, “bacterial strain”, and “microbiology”. Antibiotic resistance in non-human bacteria is a growing problem exacerbated by the irrational use of antibiotics in animals. Recent studies have highlighted the prevalence of ciprofloxacin resistance in bacteria isolated from farm animals, underscoring the need to monitor and control antibiotic use in non-human settings to prevent the spread of resistance [13]. Additionally, the development of resistance is driven by the positive selection of resistant clones, with multiple molecular mechanisms involved that reduce antibiotic effectiveness [14].
- Green Cluster: This cluster addresses the chemical components and extracts of algae, with terms such as “plant extract”, “antioxidant activity”, “algal extract”, “seaweed”, “brown algae”, and “red alga”. Algal extracts have demonstrated significant antioxidant and antimicrobial properties. Red and brown algae extracts contain bioactive compounds like polyphenols and flavonoids, which are responsible for these beneficial properties [15]. These findings highlight the potential of algal extracts in pharmaceutical and food applications, emphasizing the importance of identifying and characterizing these bioactive compounds.
- Yellow Cluster: This cluster centers on the biology of algae and the biosynthesis of bioactive compounds, with terms such as “algae”, “biosynthesis”, “microorganisms”, “metabolites”, and “cell viability”. The biosynthesis of secondary metabolites in algae offers potential applications in medicine and biotechnology. Advanced techniques like electron microscopy and infrared spectroscopy are crucial for the detailed characterization of algae structure and composition, enabling a deeper understanding of their biosynthetic processes [16]. Comparative studies between algae and other organisms like yeasts and plants provide a broad perspective on the biosynthetic capabilities of algae. Detailed analysis of cell viability and the metabolites produced can also open new avenues for innovative biotechnological applications.
- Blue Cluster: This cluster focuses on water treatment and the presence of antibiotics in aquatic environments, with key terms such as “water”, “wastewater”, “antibiotics”, “water management”, and “water pollutants, chemical”. Antibiotic contamination in wastewater and natural water bodies poses a significant risk to aquatic life, including fish and invertebrates. Antibiotics have been shown to affect the microbiome of these organisms, promoting bacterial resistance [17]. Furthermore, water management strategies, such as advanced wastewater treatment, can significantly reduce the concentration of antibiotics in water, mitigating their environmental impact.
- Purple Cluster: This cluster centers on the research of new pharmacological compounds derived from algae, with terms like “unclassified drug”, “drug structure”, “seaweed”, “bioactive compounds”, and “polyketide”. Marine algae are a rich source of bioactive compounds, including polyketides and other secondary metabolites, with significant pharmacological potential. Isolation and purification techniques, such as mass spectrometry, have identified new compounds with antibacterial and anticancer activities [18]. These findings emphasize the importance of algae as a source of new pharmacological compounds and highlight the crucial role of advanced techniques in their discovery and characterization.

### 2.3. Analysis of Temporal Trends

**Figure 3:**
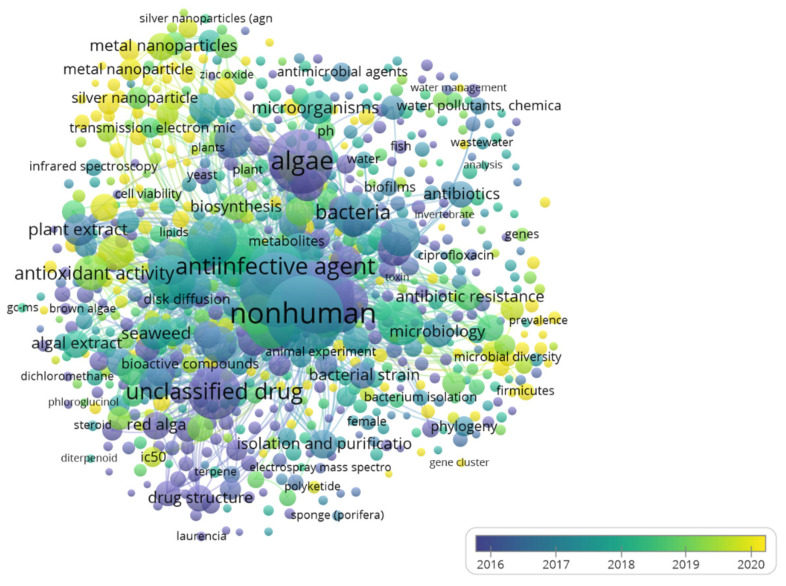
Co-occurrence map in terms of Algae-Derived Antimicrobial Research Products Over Time according to relevant scientific publications with the specific keywords: (Algae OR Meta Cyano-bacteria) and (antimicrobial OR antibiotic OR bioactive) between 2000 and 2024.

Figure 3 integrates a temporal analysis of publications revealing the evolution of research areas in algae and antimicrobials. A growing interest in antibiotic resistance is highlighted in the group related to interactions between bacteria and antibiotics, reflecting global concern. The increasing trend in antibiotic resistance research underscores the urgency of developing new strategies to mitigate this global challenge [19]. This is demonstrated by the study of Torres et al. [20], which highlights a significant increase in interest and international collaboration in this area, emphasizing the need for further research and development of effective strategies to combat this threat.

The increasing focus on algae extracts in studies of antioxidant activity reflects growing interest in the use of natural products in the pharmaceutical and food industries. Numerous investigations have shown that red and brown algae contain bioactive compounds, such as polyphenols and flavonoids, responsible for their potent antioxidant and antimicrobial properties. For instance, red algae like *Gracilaria gracilis* and *Laurencia obtusa* contain high levels of phenolic compounds and flavonoids, which significantly contribute to their antioxidant capacity and antibacterial activity [21]. Similarly, brown algae, such as *Fucus vesiculosus* and *Ascophyllum nodosum*, have shown high polyphenol content and antioxidant capacity, suggesting promising applications as functional foods and therapeutic supplements [22,23]. Recently, studies have also highlighted the potential of green algae in this field; *Chlamydomonas reinhardtii*, for example, contains sulfated polysaccharides and other bioactive metabolites with antioxidant and antibacterial activities, which are of interest in functional foods and cosmetic applications [24]. Another study on *Ch. agloeformis* identified notable antioxidant activity attributed to its phenolic compounds, expanding its potential for nutraceutical and pharmaceutical applications [25]. These findings underscore that red, brown, and green algae represent a rich source of bioactive compounds with versatile applications in the food and pharmaceutical industries. This increase in research suggests a growing demand for natural and sustainable alternatives to improve health and well-being. Regarding algae biology and compound biosynthesis, there is constant interest in significant evolution driven by their biotechnological applications [26]. In the early years, essential microbiology and molecular biology techniques predominated. However, with technological advancement, more sophisticated methodologies like electron microscopy and infrared spectroscopy have been incorporated, allowing a more detailed understanding of algae structures and compositions and facilitating the identification of their biosynthetic capacities and biotechnological potential [27].

On the other hand, the research focus has also notably shifted over time. Initially, it centered on algae’s basic biology and biosynthetic processes. Over time, increased interest has been in antibiotic resistance and antibiotic contamination in the aquatic environment [28]. Interdisciplinary collaboration has played a crucial role in the evolution of research in this field. The combination of knowledge from microbiology, chemistry, biotechnology, and environmental sciences has enriched the study, allowing for a better understanding of the problems and the development of innovative solutions [29].

Moreover, the practical applicability of research results has evolved, especially in developing new pharmacological compounds derived from algae. Identifying bioactive compounds with antibacterial and anticancer activities highlights the significant pharmacological potential of these organisms. The growing demand for natural and sustainable alternatives to improve health and well-being has driven this trend [9].

Finally, globalization and international collaboration have expanded the scope of research, allowing for the exchange of knowledge and resources worldwide. This has led to greater diversity in research and facilitated the resolution of complex problems through collaborative approaches [30]. However, despite the advances, there are biases and controversies in research, especially regarding the efficacy and safety of natural compounds. Variability in the composition of algae extracts and the lack of standardization in study methods can lead to inconsistent results. Additionally, research on antibiotic resistance can be influenced by commercial and political interests, posing ethical and transparency challenges [31].

### 2.4. Bioactive Compounds Derived from Microalgae and Cyanobacteria

Bioactive compounds derived from microalgae and cyanobacteria exhibit a remarkable diversity in their mechanisms of action and antimicrobial efficacy, granting them significant effectiveness against a wide range of pathogens. These compounds exert their antimicrobial effects through various mechanisms, including inhibition of protein synthesis, disruption of the cell membrane, and interference with bacterial metabolic processes (see Table 1). Below, these compounds’ primary mechanisms of action are detailed, explaining their functionality and distinctive characteristics.

#### 2.4.1. Compounds That Inhibit Protein Synthesis

*Oscillatoria* sp. and *Microcystis aeruginosa* produce microcystins, cyclic peptides that inhibit bacterial protein synthesis by binding to ribosomes. Microcystins act as potent inhibitors of peptide elongation during translation, disrupting the production of essential proteins critical for bacterial viability and thereby interfering with key cellular functions. Similarly, various antimicrobial peptides, such as the Bac5(1-25) fragment, demonstrate specific inhibitory activity on the bacterial ribosome, achieving high efficacy against Gram-negative bacteria without exhibiting toxicity to eukaryotic cells [32]. From a pharmacodynamic perspective, the inhibition induced by microcystins results in a rapid reduction in bacterial cell viability; a similar effect is observed with the defense peptide Hylin-a1, which is highly effective at low concentrations and exhibits activity against antibiotic-resistant *S. aureus* strains [33]. Additionally, the bioavailability of microcystins may vary based on formulation and administration method, enabling optimization of stability and duration to maximize antimicrobial efficacy [34].

Moreover, certain antimicrobial peptides, such as Esc(1-21)-1c, have shown synergy when combined with conventional antibiotics, enhancing the susceptibility of *P. aeruginosa* through inhibition of bacterial efflux pump expression [35]. This synergistic combination of AMPs and antibiotics presents a promising strategy against multidrug-resistant bacteria, as it increases antimicrobial efficacy and helps to overcome resistance barriers [36]. These peptides have also demonstrated anti-biofilm properties on medical devices, making them a valuable preventive and therapeutic tool for infections associated with surfaces [37].

In addition to previously described mechanisms, several studies have demonstrated the high efficacy of these compounds at low inhibitory concentrations, underscoring their potential as antimicrobial agents. [38] reported that microcystins from *M. aeruginosa* have a minimum inhibitory concentration (MIC) ranging between 0.5 and 1.0 μg/mL against *S. aureus*, demonstrating significant antimicrobial activity at minimal concentrations. Similarly, [39] noted that these compounds may facilitate the transfer of resistance genes among bacteria, suggesting a regulatory role in maintaining microbial balance in the environment. Furthermore, [40] reported that certain Antarctic strains inhibited *M. aeruginosa* with MICs between 0.55 and 3.00 mg/mL, which opens possibilities for their use in biocontrol. Additionally, [41] highlighted that the Bac5(1-25) peptide inhibits bacterial ribosomes in Gram-negative bacteria with an MIC of 1 μM, demonstrating low toxicity in eukaryotic cells and effectiveness against resistant bacteria. These experimental findings reinforce the relevance of protein synthesis inhibitors in developing effective antimicrobial agents.

On the other hand, *Synechococcus* sp. produces antimicrobial peptides that inhibit bacterial protein synthesis, acting similarly to traditional antibiotics such as tetracycline. These peptides demonstrate a high affinity for bacterial ribosomes, blocking protein synthesis. Pharmacodynamically, they are effective at low concentrations, with rapid action against multiple bacterial strains. Pharmacokinetically, these peptides exhibit good stability in biological media and an appropriate half-life for therapeutic applications. Additionally, Anabaena sp. produces anabaenopeptin, another peptide that inhibits protein synthesis. Mendes et al. [42] showed that anabaenopeptin is effective against Gram-positive and Gram-negative bacteria, indicating its utility in developing new antimicrobial agents. Pharmacodynamically, anabaenopeptin has a bacteriostatic effect, halting bacterial growth without necessarily killing the cells. Its absorption and distribution profile are favorable pharmacokinetically, allowing prolonged action at the infection site. Finally, *Pseudanabaena* sp. produces pseudanabaenapeptin, which also inhibits protein synthesis. Plaza et al. [43] highlight that this compound is effective against various bacteria, making it a promising candidate for antimicrobial applications. Pharmacodynamically, pseudanabaenapeptin blocks protein elongation, while pharmacokinetically, its formulation allows sustained release and effective penetration into target tissues.

#### 2.4.2. Compounds That Disrupt the Cell Membrane

*Chlorella* sp. and *Nannochloropsis oculata* are sources of polyunsaturated fatty acids such as α-linolenic acid and Eicosapentaenoic Acid (EPA), respectively. These fatty acids disrupt the bacterial cell membrane, resulting in cell lysis. Hussein et al. [38] demonstrated that these compounds are notably effective against Gram-negative bacteria. Pharmacodynamically, the insertion of these fatty acids into the bacterial membrane increases its fluidity and permeability, causing cell death. Pharmacokinetically, these fatty acids integrate well into biological membranes and can be formulated in various delivery systems to enhance their bioavailability and stability.

Similarly, *Picochlorum* sp. produces palmitic acid, disrupting the bacterial cell membrane. Hussein et al. [38] found that palmitic acid is effective against Gram-negative bacteria, highlighting its potential in antimicrobial treatments. Pharmacodynamically, palmitic acid destabilizes the bacterial membrane structure, leading to cell lysis. Pharmacokinetically, palmitic acid shows good absorption and uniform distribution in tissues, with efficient elimination from the organism.

Complementing these findings, several studies highlight the effectiveness of compounds that disrupt bacterial membrane integrity at practical concentrations. For instance, α-linolenic acid derived from *Chlorella* sp. showed a minimum inhibitory concentration (MIC) of 10 μg/mL against *E. coli*, promoting rapid cell lysis [38]. [44] also found that *B. licheniformis* exerts algicidal activity against *M. aeruginosa* through lipid peroxidation, compromising membrane structure. Moreover, [45] observed that biomimetic polymers designed to mimic lipid components inhibit the proliferation of *Ch. reinhardtii* and *Sc. elongatus* with MICs ranging from 95 nM to 6.5 μM, interacting directly with cellular membranes. These results strengthen the evidence that lipid-derived compounds from microalgae significantly impact bacterial membrane integrity, making them promising agents for targeted antimicrobial treatments.

#### 2.4.3. Compounds with Varied Mechanisms

In addition to the compounds mentioned above, *Arthorspira* sp. produces C-phycocyanin, a pigment protein that inhibits the growth of various bacteria, demonstrating a broad spectrum of antibacterial activity. Jyotirmayee et al. [46] found that this compound could help prevent infections, especially in clinical settings where bacterial resistance is a growing problem. C-phycocyanin acts as a bacterial growth inhibitor and possesses anti-inflammatory and antioxidant properties, which could benefit combined treatments. Pharmacodynamically, C-phycocyanin inhibits key enzymes in inflammatory and oxidative processes, while pharmacokinetically, it distributes well in the body and has an adequate half-life for sustained therapeutic effects.

Furthermore, *Phormidium autumnale* contains acids such as gentisic acid, vanillic acid, and p-coumaric acid, which have proven effective against methicillin-resistant *S. aureus* (MRSA) and other pathogens. Yalcin et al. [47] highlight that these compounds can be valuable in treating infections resistant to traditional antibiotics, offering a new therapeutic pathway. Combining these acids provides a synergistic mechanism of action that enhances their antimicrobial efficacy. Pharmacodynamically, these acids interfere with cell wall synthesis and membrane function, while pharmacokinetically, they exhibit good tissue penetration and efficient elimination, improving their therapeutic profile.

In addition to the mechanisms described, several studies have documented the efficacy of compounds with varied mechanisms against different types of pathogens. [47] reported that gentisic acid derived from *Phormidium autumnale* effectively inhibits the growth of methicillin-resistant *S. aureus* (MRSA) with a minimum inhibitory concentration (MIC) of 5 μg/mL, highlighting its potential for resistant bacterial infections. [48] also found that derivatives of ursolic acid exhibit significant antibiofilm activity against *Acinetobacter baumannii*, with MICs ranging from 78 to 156 μg/mL, supporting its application in infections involving biofilm formation. Furthermore, [49] demonstrated that fucoidans extracted from Fucus vesiculosus inhibit Escherichia coli growth with MICs of 4–6 mg/mL, indicating effective antibacterial activity. Collectively, these studies suggest that microalgal compounds with diverse mechanisms offer versatile antimicrobial solutions applicable to complex infections and multidrug-resistant pathogens. Similarly, *Tetraselmis suecica* and *Dunaliella salina* produce fucose sulfate and beta-carotene, respectively. Fucose sulfate interferes with bacterial cell adhesion, while beta-carotene has antioxidant properties. Both compounds inhibit bacterial growth and protect against oxidative damage, as observed by Borowitzka [43]. These properties suggest potential applications in developing combined treatments to address multiple aspects of infection and inflammation. Pharmacodynamically, fucose sulfate prevents biofilm formation, and beta-carotene neutralizes free radicals, while pharmacokinetically, both compounds show good absorption and distribution in the body.

Additionally, *Euglena viridis* produces clofibric acid, which interferes with lipid metabolism, effectively reducing lipid levels and bacteria. Jyotirmayee et al. [46] suggested that this compound could have therapeutic applications in both bacterial infections and metabolic conditions, offering a dual approach to treatment. Clofibric acid acts against bacteria and improves the host’s metabolic conditions, making it valuable in treating infections complicated by metabolic disorders. Pharmacodynamically, clofibric acid reduces lipid synthesis and destabilizes the bacterial membrane, while pharmacokinetically, it distributes widely and is efficiently eliminated. Finally, *Cryptomonas* sp. produces crocin, a carotenoid that inhibits bacterial growth and has demonstrated efficacy against bacteria and fungi. Borowitzka [43] suggests that this compound can be valuable in developing new antimicrobials with applications in mixed infections. Crocin has antimicrobial properties and protects against oxidative stress, enhancing its potential therapeutic benefits. Pharmacodynamically, crocin inhibits vital enzymes involved in microbial metabolism, while pharmacokinetically, it shows good bioavailability and a favorable half-life for sustained antimicrobial action.

**Table 1:** Antimicrobial Compounds from Microalgae and Cyanobacteria.

Organism	Identified Antimicrobial	Mechanism of Action	Reference
*Anabaena* sp.	Anabaenopeptin	Inhibits protein synthesis	[42]
*Arthorspira* sp.	C-Phycocyanin	Inhibits bacterial growth	[46]
*Chlorella* sp.	α-Linolenic acid	Membrane permeabilization	[38]
*Cryptomonas* sp.	Crocin	Inhibits bacterial growth	[43]
*Dunaliella salina*	Beta-carotene	Antioxidant action	[43]
*Euglena viridis*	Clofibric acid	Interferes with lipid metabolism	[46]
*Microcystis aeruginosa*	Microcystin	Inhibits protein synthesis	[50]
*Nannochloropsis oculata*	EPA (Eicosapentaenoic Acid)	Alters cell membrane	[38]
*Nostoc* sp.	Nostopeptolide	Inhibits protein synthesis	[42]
*Oscillatoria* sp.	Microcystin	Inhibits protein synthesis	[50]
*Phormidium autumnale*	Gentisic acid, vanillic acid, p-coumaric acid	Inhibits bacterial growth	[47]
*Picochlorum* sp.	Palmitic acid	Membrane permeabilization	[38]
*Pseudanabaena* sp.	Pseudanabaenapeptin	Inhibits protein synthesis	[43]
*Synechococcus* sp.	Antimicrobial peptides	Inhibits protein synthesis	[43]
*Tetraselmis suecica*	Fucosaquosa	Interferes with cell adhesion	[38]

### 2.5. Antimicrobial Efficacy of Compounds Derived from Microalgae and Cyanobacteria

The efficacy of antimicrobial compounds derived from microalgae and cyanobacteria has proven remarkable against many pathogens. These compounds not only inhibit the growth of common bacteria but are also effective against multidrug-resistant strains, making them promising candidates for developing new antimicrobial treatments [43] (Table 2). These compounds’ structural diversity and unique mechanisms of action contribute to their broad efficacy. To evaluate their true clinical potential, it is essential to consider not only the compounds’ ability to inhibit bacterial growth but also their spectrum of action, the dosage required to achieve therapeutic effects, and their capacity to prevent the development of bacterial resistance. Additionally, it is essential to analyze the stability of these compounds, their bioavailability in the body, and their potential toxicity and side effects. Among the most important compounds are microcystins, nostopeptolide, and antimicrobial peptides from *Synechococcus* sp. and *Anabaena* sp.

Microcystins, produced by *Oscillatoria* sp. and *Microcystis aeruginosa*, exhibit high specificity and potency, acting rapidly against bacteria such as *S. aureus* and *Pseudomonas aeruginosa*. The main advantage of these substances lies in their ability to inhibit protein synthesis by binding to bacterial ribosomes, resulting in a rapid decrease in bacterial cell viability. However, their potential toxicity to human cells and high production costs limits their immediate clinical applicability [50]. Similarly, nostopeptolide, an antimicrobial peptide produced by *Nostoc* sp., is highly effective at low concentrations and inhibits protein synthesis. Its efficacy against *Escherichia coli* and *S. aureus* positions it as a promising agent (Table 2).

Nevertheless, additional studies on its pharmacokinetics and metabolism present challenges for clinical development [42]. Moreover, the antimicrobial peptides from *Synechococcus* sp. and *Anabaena* sp. offer a broad spectrum of action and high affinity for bacterial ribosomes, effectively inhibiting protein synthesis in Gram-positive and Gram-negative bacteria. Despite their efficacy, the potential development of resistance and specific storage requirements represent significant challenges for their long-term use [43].

Alpha-linolenic acid from *Chlorella* sp. and Eicosapentaenoic Acid (EPA) from *N. oculata* also show high efficacy, altering the bacterial cell membrane and resulting in cell lysis. These fatty acids are particularly effective against Gram-negative bacteria and prevent biofilm formation, which is essential for treating persistent infections. However, their variability in efficacy and stability issues, such as the potential for oxidation and degradation, require innovative solutions in formulation and storage [38]. Similarly, palmitic acid from *Picochlorum* sp. destabilizes the cell membrane, demonstrating efficacy against Gram-negative bacteria. Although it is a promising compound, its efficacy is limited to Gram-negative bacteria, and the need for specific formulations to improve its stability and clinical effectiveness is a significant challenge [38].

Furthermore, C-phycocyanin, a pigment protein from cyanobacteria and red algae, inhibits the growth of various bacteria and offers anti-inflammatory and antioxidant properties, which can be beneficial in combined treatments. However, its potential toxicity at high doses and production costs are significant drawbacks requiring commercial viability attention [46]. Additionally, organic acids such as gentisic acid, vanillic acid, and p-coumaric acid from *Ph. autumnale* are effective against resistant pathogens like methicillin-resistant *S. aureus* (MRSA) and other pathogens, thanks to their synergistic action that destabilizes the cell membrane and affects cell wall synthesis. Nevertheless, the need for more clinical studies and variability in efficacy present significant challenges for their clinical implementation [47]. Clofibric acid from *Euglena viridis* is notable for its effectiveness in reducing lipid levels and bacteria, offering additional benefits in treating infections complicated by dyslipidemias. However, it requires optimization in its formulation and presents potential toxicity at high doses, limiting its direct application [46]. Lastly, crocin from *Cryptomonas* sp., a carotenoid with antioxidant and anti-inflammatory properties, is effective against bacteria and fungi. Despite its great potential, the need for more studies on its stability and formulation is a significant barrier to its development and clinical application [43].

### 2.6. Purification of Bioactive Compounds

The extraction and purification of bioactive compounds derived from microalgae and cyanobacteria are fundamental processes for obtaining high-quality products suitable for pharmaceutical and other industrial applications. The most used methods of these processes include solvent extraction, supercritical extraction, and various separation techniques, such as chromatography. The extraction and purification method choice depends on the type of bioactive compound and factors such as efficiency, environmental impact, and associated costs. Each method has its specific advantages and disadvantages, and ongoing research is essential to optimize these processes and make them more viable for large-scale production (Table 3).

- Solvent extraction is a widely used method that employs organic solvents to extract bioactive compounds from biomass. This method is highly effective in recovering compounds, including lipids, proteins, and pigments. However, it presents particular challenges, such as high costs and environmental concerns due to the waste generated during the process. According to El-Sapagh et al. [51], although solvent extraction is efficient, its sustainability is questionable due to the associated environmental impacts.
- Supercritical extraction uses carbon dioxide in a supercritical state to extract bioactive compounds. This method offers a cleaner and more efficient alternative than traditional solvent extraction. Supercritical carbon dioxide acts as a solvent that can penetrate the biomass and dissolve bioactive compounds without leaving toxic residues. Mendes et al. [52] demonstrated that supercritical extraction is highly efficient and environmentally friendly, although it requires specialized equipment and is more expensive for initial investment and operation.
- Separation techniques, such as chromatography, are essential for purifying the extracted bioactive compounds, ensuring the acquisition of high-purity products suitable for pharmaceutical applications. Chromatography allows the separation of compounds based on their chemical and physical properties, such as polarity and molecular weight. Plaza et al. [53] evidenced that chromatography efficiently obtains high-purity compounds. However, the costs associated with these techniques can be prohibitive on a large scale due to the need for advanced equipment and specialized consumables.

**Table 3:** Comparison of Methods for Extraction and Purification of Bioactive Compound.

Bioactive Compound	Most Used Method	Advantages	Disadvantages	Reference
Alpha-linolenic Acid	Solvent Extraction	Efficient lipid recovery	Environmental issues	[51]
Antimicrobial Peptides	Chromatography	High purity of the final product	Prohibitive costs at large scale	[53]
C-phycocyanin	Chromatography	High-purity products	High costs	[53]
Clofibric Acid	Solvent Extraction	High efficiency	Sustainability issues	[51]
Crocin	Chromatography	High purity for pharmaceutical applications	Prohibitive costs	[53]
EPA (Eicosapentaenoic Acid)	Supercritical Extraction	Efficient extraction without toxic residues	High initial investment	[52]
Microcystins	Solvent Extraction	High extraction efficiency	Environmental and sustainability issues	[51]
Nostopeptolide	Supercritical Extraction	Cleanliness and efficiency	High costs and need for specialized equipment	[52]
Organic Acids	Supercritical Extraction	Sustainable and efficient methods	Requires advanced technology	[52]
Palmitic acid	Solvent Extraction	Proven and established method	Environmental impact	[51]

### 2.7. Technological and Economic Optimization in the Production of Algae-Derived Antimicrobial Agents

Producing antimicrobial agents from microalgae is an innovative and sustainable approach that requires careful optimization of cultivation and processing factors to maximize efficiency. Variables such as reactor type, nutrient availability, light, pH, and temperature play critical roles in bioactive compound synthesis and biomass yield. For industrial-scale application of these technologies, it is essential to consider both environmental factors and production system characteristics to create optimal conditions for the growth of microalgae like *Ch. reinhardtii* and other species with documented antimicrobial potential.

#### 2.7.1. Reactor Type

Microalgae cultivation reactors include both open and closed systems, each with distinct characteristics and applications. Open systems, such as ponds and lagoons, are economically viable and support large-scale biomass production but are vulnerable to environmental variations and contamination risks. Studies on *Ch. reinhardtii* have shown promising results for antimicrobial production in open systems, provided that management practices are implemented to minimize environmental variability and contaminant levels, despite some limitations in product stability [54]. In *C. vulgaris* and *Botryococcus braunii*, open reactors yielded satisfactory levels of biomass and bioactive compounds, though with increased maintenance requirements to optimize output and control light and temperature conditions [55]. Closed reactors, such as tubular and flat-plate photobioreactors, enable precise control of cultivation parameters, enhancing antimicrobial production and final product quality. A tubular photobioreactor used with *Ch. reinhardtii* improved antimicrobial production efficiency by adjusting CO_2_ and light, maintaining high stability of the obtained bioactive compounds [56]. In *Nannochloropsis oculata*, these systems achieved energy efficiencies up to 20 W/m^3^, providing superior benefits for bioactive compound production over open systems, thanks to tighter temperature and light control [57].

#### 2.7.2. Nutrients

Nutrients, particularly nitrogen and phosphorus, are essential for lipid synthesis and antimicrobial metabolite production in microalgae. In *C. pyrenoidosa*, regulating nitrate at 16.8 mM and phosphate at 300.9 µM increased lipid production up to 2.9 times, essential for antimicrobial properties [58]. In *Ch. reinhardtii*, nitrogen-rich effluent improved the synthesis of antimicrobial exopolysaccharides, particularly in mixed culture applications [59]. Other studies on *Sc. obliquus* have shown that balanced nitrogen and phosphorus concentrations are critical for sustained cell growth, ensuring continuous bioactive metabolite production [55]. Carbon sources like CO_2_ and organic compounds have also significantly boosted antimicrobial biomass. In *C. vulgaris*, 0.5% CO_2_ combined with glycerol increased biomass by 45%, optimizing antimicrobial compound synthesis [60], while in *Ch. reinhardtii*, high CO_2_ levels promoted glycerol accumulation, enhancing the production of bioactive antimicrobials [57].

#### 2.7.3. Light (Intensity, Wavelength, and Photoperiod)

Light intensity, wavelength, and photoperiod significantly impact antimicrobial metabolite synthesis. In *Ch. reinhardtii*, exposure to a 12-h light and 60-h dark photoperiod optimized antimicrobial production, improving the efficacy of generated compounds against pathogenic bacteria [61]. In *Nannochloropsis* sp., blue light wavelength increased antimicrobial lipid production, while in Scenedesmus, adjusting light intensity and wavelength enhanced antioxidant and antimicrobial metabolite production, highlighting the importance of tailoring light conditions to maximize bioactive compound output [62,63].

#### 2.7.4. pH and Temperature

Maintaining optimal pH and temperature is crucial for stable antimicrobial production in microalgae. In *C. vulgaris*, a pH of 9.0 combined with a temperature of 25 °C maximized antimicrobial lipid synthesis, while in *Ch. reinhardtii*, the antimicrobial compound Mytichitin-A showed high stability across broad pH and temperature ranges, adapting effectively to variable conditions [60,64]. Studies on *A. platensis* found that temperatures between 30 and 35 °C favored antimicrobial compound production, underscoring the importance of thermal control for optimal bioactive synthesis efficiency [62].

#### 2.7.5. Economic and Energy Sustainability

The production of antimicrobial agents from microalgae presents a viable and sustainable alternative to conventional methods, particularly when strategies are implemented to optimize nutrient use and improve harvesting and processing systems. To maintain the purity of antimicrobial compounds, it is essential to rigorously control nutrient supply. Recent studies indicate that *Ch. reinhardtii* significantly improved antimicrobial metabolite production with adjusted nitrogen and phosphorus levels, reducing waste accumulation and preserving the efficacy of the final product [65]. Furthermore, in *Sc. obliquus* cultures, controlled administration of phosphate and iron proved critical for achieving high biomass productivity without compromising antimicrobial quality, emphasizing the importance of managing each nutrient individually [66].

In addition, reducing operational costs through advanced harvesting techniques has been a crucial focus. Electroflotation, a technique that enables biomass collection without chemical additives, has shown high efficacy. In *Ch. reinhardtii*, this method achieved collection efficiencies exceeding 90%, which is fundamental for preserving antimicrobial activity [67]. Similarly, studies on *Sc. obliquus* confirmed that electroflotation reduces energy consumption compared to traditional methods, establishing it as a sustainable and cost-effective option for antimicrobial applications [68].

Simultaneously, controlled CO_2_ use in closed culture systems contributes to both biomass growth and the quality of the final antimicrobial compound. Research with *Ch. reinhardtii* showed that administering CO_2_ under controlled conditions improved growth efficiency without affecting antimicrobial activity, underscoring CO_2_’s potential as a sustainable resource in this context [69]. Complementary studies with *C. vulgaris* revealed that continuous CO_2_ supply not only enhanced photosynthetic efficiency but also reduced the need for other nutrients, thus contributing to the economic sustainability of the process [70]. Nutrient recycling in closed-loop systems offers an effective strategy to reduce operational costs without compromising antimicrobial purity. In *C. vulgaris*, nitrogen and phosphorus recovery in a closed system reduced input costs by up to 40% while maintaining the quality of the final compound [71]. Moreover, the use of photobioreactors in *Ch. reinhardtii* cultures optimized nutrient recovery and lowered emissions, reinforcing the economic and environmental feasibility of producing antimicrobials from microalgae [72].

To enhance the stability and durability of algae-derived antimicrobial agents under unfavorable environmental conditions, the use of nanotechnology—particularly encapsulation techniques—has proven highly effective. Encapsulation within nanoparticles, such as those composed of poly(lactic-co-glycolic acid) (PLGA), ensures the antimicrobial agents are protected, enabling controlled release and significantly reducing degradation during storage and processing. [38] observed that encapsulating antimicrobial peptides in PLGA not only improved stability but also allowed for a reduction in the minimum inhibitory concentration against *S. aureus*, thereby enhancing antimicrobial efficacy. Similarly, [73] found that cationic-coated shellac nanocapsules improved the release of encapsulated berberine and increased capsule interaction with the bacterial cell wall, thereby augmenting its antibacterial potency. Moreover, [74] evaluated the benefits of encapsulation in nanomatrices, noting enhanced stability and shelf life of antimicrobial compounds by reducing oxidation and degradation during storage—an essential aspect for pharmaceutical and food applications. These advanced encapsulation techniques are proving critical for improving the stability and efficacy of algae-derived antimicrobials across various industrial applications.

To ensure that antimicrobial agents derived from microalgae maintain consistent quality and efficacy, implementing quality control protocols for each production batch is essential. Advanced tools such as liquid chromatography coupled with mass spectrometry (LC-MS) and infrared spectroscopy enable precise monitoring of the purity and concentration of bioactive compounds, ensuring each batch meets efficacy and safety standards. In food applications, [41] demonstrated that encapsulating antimicrobials in nanostructures, such as nanoemulsions and liposomes, not only maintains antimicrobial activity over time but also significantly enhances stability during extended storage. [75] emphasized that nanoencapsulation allows greater standardization of antimicrobial compounds, minimizing batch-to-batch variability and ensuring consistent quality in the final product. Combining these encapsulation techniques with standardized quality controls not only ensures that microalgae-derived antimicrobials comply with regulatory standards but also enhances their commercial viability in pharmaceutical and food sectors, positioning these compounds as safe, effective, and sustainable alternatives in an increasingly competitive market.

## 3. Future Perspectives

Despite significant advances, several challenges must still be addressed to fully realize the potential of natural antimicrobial products derived from algae. A primary issue is the variability in the chemical composition of algae, influenced by environmental factors, complicating the standardization and reproducibility of bioactive compounds and making it difficult to ensure consistent product quality [76]. These compounds’ extraction and purification processes can also be complex and costly, limiting their commercial application [77].

Genetic engineering has enabled significant improvements in the production of antimicrobial compounds in microalgae through a range of advanced genetic editing tools. In addition to CRISPR-Cas9, techniques such as TALEN (transcription activator-like effector nucleases) and ZFN (zinc finger nucleases) have been successfully employed for targeted modifications in *Ch. reinhardtii*. [78] highlights the use of CRISPR-Cas9 to achieve specific knock-ins and knock-outs in genes involved in metabolic pathways for antimicrobial production, achieving a 37% increase in editing efficiency by optimizing sgRNA (single-guide RNA) sequences. Additionally, [79] induced the overexpression of heterologous genes in *Chlamydomonas* sp. using CRISPR-Cas9 in combination with specific insertions, enhancing the production of high-value antimicrobial metabolites. Furthermore, the CRISPR-Cpf1 system, which offers advantages in removing unwanted sequences from the genome of *Anabaena* sp. PCC 7120, was employed by [80] for rapid and effective editing at multiple loci, expanding the engineering potential in microalgae for antimicrobial applications. These advanced tools not only optimize antimicrobial production but also address challenges related to the adaptation and stability of microalgae in large-scale cultivation conditions.

To tackle these challenges, various solutions have been proposed. One is advanced cultivation systems, such as integrated biorefineries, which can enhance yields and reduce production costs [81,82]. Moreover, algae’s genetic and metabolic engineering can increase the production of specific bioactive compounds and enable the creation of new compounds through combinatorial biosynthesis. These approaches improve the output of existing compounds and facilitate the optimization of metabolic pathways, opening new opportunities for developing innovative antimicrobial agents. Genetic and metabolic engineering of algae allows the manipulation of biosynthetic pathways to increase the production of specific compounds and create new compounds through combinatorial biosynthesis. This approach has been highlighted in recent studies, where manipulating critical genes in specific metabolic pathways has produced new metabolites with enhanced antimicrobial activities [83]. The application of advanced techniques such as CRISPR gene editing and synthetic biology has allowed for optimizing the production of bioactive compounds in algae, representing a significant advance in natural antimicrobial products [84]. However, genetic modification of algae raises important ethical and environmental considerations. The release of genetically modified organisms into natural ecosystems can have unforeseen ecological impacts, necessitating comprehensive risk assessments and containment strategies [85,86].

Furthermore, it is essential to develop eco-friendly and efficient methods for extracting and purifying bioactive compounds, making these compounds more accessible for industrial and therapeutic applications. Innovations such as pressurized liquid extraction, ultrasound, microwave, and high-intensity pulsed electric fields have shown promise [12]. Additionally, integrating nanotechnology can enhance bioactive compounds’ delivery and efficacy. These advanced technologies optimize extraction and ensure the preservation of phenolic structures and other bioactive properties of the compounds, which is crucial for maintaining their antimicrobial effectiveness [11].

Extensive clinical trials are crucial for validating the efficacy and safety of these compounds in humans and developing new delivery systems to improve the bioavailability and efficacy of bioactive compounds [87]. Preclinical and clinical validation of bioactive compounds is fundamental for their therapeutic implementation. In vivo studies must evaluate the compounds’ pharmacokinetics, toxicity, and efficacy, while clinical trials must confirm their safety and effectiveness in humans [88]. Preclinical evaluation is crucial for determining how compounds behave in a living organism, providing data on bioactive compounds’ absorption, distribution, metabolism, and excretion (ADME). Recent studies have highlighted the importance of these trials, noting that many promising compounds in vitro fail to demonstrate efficacy in animal models or clinical trials due to issues with bioavailability or toxicity [89]. Conducting rigorous preclinical studies followed by well-designed clinical trials is essential for translating laboratory discoveries into safe and effective human therapies [90]. However, conducting these studies is resource-intensive and time-consuming, presenting significant obstacles to the rapid advancement of these products [91].

The assertion that these products have “enormous potential to address antimicrobial resistance” is supported by evidence showing their effectiveness against various pathogens. However, significant challenges must be overcome to realize this potential, including standardization, large-scale production, and regulatory approval. Standardization ensures that each product batch contains the same number of bioactive compounds, a challenge due to the inherent variability in natural raw materials. Large-scale production also presents technical and economic difficulties, as efficient and sustainable cultivation and extraction methods must be developed to handle large volumes of algal biomass. Additionally, regulatory approval requires extensive testing to demonstrate the product’s safety and efficacy, which can be lengthy and costly. Recent studies have highlighted these challenges, discussing the barriers and strategies for commercializing algae-derived products [92]. Overcoming these challenges requires interdisciplinary collaboration and the development of innovative technologies to ensure the quality and sustainability of algae-derived products [93]. While promising, these products are still in the early stages of development, and their full potential will only be realized through continued research and interdisciplinary collaboration [94].

## 4. Materials and Methods

The Scopus database (https://www.scopus.com accessed on 10 October 2024) was selected for the bibliometric analysis due to its broad coverage of relevant scientific publications. Specific keywords were used: (Algae OR Meta Cyanobacteria) AND (antimicrobial OR antibiotic OR bioactive), to cover traditional and advanced methods in antimicrobial natural products research. The analysis was performed using VOSviewer software (Version 1.6.20), which allowed the examination of keyword co-occurrence and visualization of bibliometric networks of publications. Articles with a minimum of 12 occurrences of the keywords in abstracts, titles, or keywords were included, and the analysis period spanned from 2000 to 2024 to capture the evolution over the last two decades. In addition, the selected files were exported as plain text, which simplified the analysis by not requiring individual review of each file. In addition, a keyword co-occurrence network was developed, and the temporal evolution was analyzed, identifying frequent terms and their interconnections. In this sense, prominent nodes such as “antibiotic resistance”, “algal extracts”, “biosynthesis”, and “water treatment” were visualized, highlighting their fundamental role in the research of antimicrobial compounds. In addition, the progression of publications over time was studied to identify emerging trends and transformations. Finally, the primary research topics and subtopics around algae-derived antimicrobial compounds were comprehensively evaluated, considering their activity and efficiency.

## 5. Conclusions

The bibliometric analysis revealed distinctive research areas, highlighting the relevance of microalgae and cyanobacteria-derived products in the pharmaceutical and food industries. Likewise, co-occurrence analysis identified key themes and interrelated connections, highlighting the central role of algae in addressing antibiotic resistance. However, several challenges must be overcome to fully exploit these bioactive compounds’ potential. First, variability in chemical composition due to environmental factors complicates the standardization and reproducibility of these compounds; therefore, it is crucial to develop strategies to mitigate this variability through specific examples. In addition, extraction and purification processes are complex and expensive, limiting large-scale production and commercial viability. In this sense, describing technological innovations that simplify and make these processes cheaper could help to overcome these barriers. On the other hand, the lack of clinical studies restricts the immediate therapeutic application of these compounds.

Future research efforts should focus on improving algal biomass production, implementing advanced cultivation systems, and applying genetic engineering to increase yields and reduce costs. Optimizing existing purification methods by developing sustainable and efficient processes will ensure the availability of high-quality products. In parallel, conducting comprehensive preclinical and clinical studies is crucial to validate the safety and efficacy of these compounds in humans. In addition, identifying new emerging research areas and proposing innovative methodologies, such as using artificial intelligence technologies to optimize cultivation and extraction, will open new opportunities in this field. Finally, interdisciplinary collaboration and public-private partnerships will be vital to advance in this area. In this context, mapping critical players in the industry and proposing specific mechanisms to foster cooperation can accelerate the development of algae-derived antimicrobial agents. Similarly, suggesting successful collaboration models in other sectors and applying circular economy principles to algae production and processing will maximize sustainability and efficiency. Finally, formulating public policies and regulations that support the development of the algae industry, together with education and awareness initiatives, will increase the knowledge and acceptance of these products among consumers and industry.

## Figures and Tables

**Table 2:** Comparative Overview of Antimicrobial Compounds from Microalgae and Cyanobacteria.

Organism	Compound	Advantages	Disadvantages	Efficacy	Reference
*M. aeruginosa*	Microcystins	High specificity and potency	Potential toxicity to human cells	*S. aureus*	[50]
		Fast action	high production cost	*P. aeruginosa*	
*Nostoc* sp.	Nostopeptolide	High effectiveness at low concentrations	Needs more studies on pharmacokinetics and metabolism	*E. coli*	[42]
		specific action		*S. aureus*	
*Synechococcus* sp.	Antimicrobial peptides	A broad spectrum of action	Possible development of resistance	Gram-positive and Gram-negative bacteria	[43]
		high affinity for ribosomes	specific storage requirements		
*Chlorella* sp.	α-Linolenic acid	Destroys bacteria	Variability in efficacy	Gram-negative bacteria	[38]
		prevents biofilms			
		A broad spectrum of action	stability issues		
*N. oculata*	EPA (Eicosapentaenoic Acid)	Effective against Gram-negative bacteria	Potential for oxidation and degradation	*E. coli*	[38]
		prevents biofilms	specific storage requirements	*S. aureus*	
*Picochlorum* sp.	Palmitic acid	Effective against Gram-negative bacteria	Limited to Gram-negative bacteria	Gram-negative bacteria	
		Destabilizes membranes	requires specific formulation		

## Data Availability

Not applicable.
